# *GHSR* DNA hypermethylation is a common epigenetic alteration of high diagnostic value in a broad spectrum of cancers

**DOI:** 10.18632/oncotarget.2759

**Published:** 2015-02-21

**Authors:** Evgeny A. Moskalev, Pouria Jandaghi, Mahdi Fallah, Mehdi Manoochehri, Sandeep K. Botla, Oleg V. Kolychev, Evgeny A. Nikitin, Vladymyr V. Bubnov, M. von Knebel Doeberitz, Oliver Strobel, Thilo Hackert, Markus W. Büchler, Nathalia Giese, Andrea Bauer, Thomas Muley, Arne Warth, Peter Schirmacher, Florian Haller, Jörg D. Hoheisel, Yasser Riazalhosseini

**Affiliations:** ^1^ Functional Genome Analysis, Deutsches Krebsforschungszentrum (DKFZ), 69120 Heidelberg, Germany; ^2^ Molecular Genetic Epidemiology, Deutsches Krebsforschungszentrum (DKFZ), 69120 Heidelberg, Germany; ^3^ Military Training Research Center, Zhukovsky – Gagarin Air Force Academy, 394064 Voronezh, Russia; ^4^ Molecular Haematology, National Research Centre for Haematology, 125167 Moscow, Russia; ^5^ Department of Genomics and Immunology, Odessa State Medical University, 265026 Odessa, Ukraine; ^6^ Department of Applied Tumor Biology, Institute of Pathology, University Hospital Heidelberg, 69120 Heidelberg, Germany; ^7^ Department of Surgery, University Hospital Heidelberg, 69120 Heidelberg, Germany; ^8^ Translational Research Unit, Thoraxklinik Heidelberg at Heidelberg University, Heidelberg, Germany; ^9^ Institute of Pathology, University Hospital, 69120 Heidelberg, Germany; ^10^ Diagnostic Molecular Pathology, Institute of Pathology, Friedrich-Alexander University Erlangen-Nürnberg (FAU), 91054 Erlangen, Germany; ^11^ Department of Translational Pneumology, Translational Lung Research Centre Heidelberg (TLRC-H), Member of the German Centre for Lung Research (DZL), 69120 Heidelberg, Germany; ^12^ Current address: Department of Human Genetics and McGill University and Genome Quebec Innovation Centre, H3A 0G1 Montreal, Quebec

**Keywords:** DNA methylation, Cancer, Diagnosis, *GHSR*, Epigenetics

## Abstract

Identification of a single molecular trait that is determinant of common malignancies may serve as a powerful diagnostic supplement to cancer type-specific markers. Here, we report a DNA methylation mark that is characteristic of seven studied malignancies, namely cancers of lung, breast, prostate, pancreas, colorectum, glioblastoma and B cell chronic lymphocytic leukaemia (CLL) (*n* = 137). This mark was defined by substantial hypermethylation at the promoter and first exon of *growth hormone secretagouge receptor* (*GHSR*) through bisulfite pyrosequencing. The degree of aberrant methylation was capable of accurate discrimination between cancer and control samples. The highest sensitivity and specificity of cancer detection was achieved for cancers of pancreas, lung, breast and CLL yielding the area under the curve (AUC) values of 1.0000, 0.9952, 0.9800 and 0.9400, respectively. Narrowing to a single CpG site within the gene's promoter or four consecutive CpG units of the highest methylation levels within the first exon improved the detection power. *GHSR* hypermethylation was detected already at the early stage tumors. The accurate performance of this marker was further replicated in an independent set of pancreatic cancer and control samples (*n* = 78). These findings support the candidature of *GHSR* methylation as a highly accurate pan-cancer marker.

## INTRODUCTION

When detected early, common cancers are often successfully controlled by current therapies, emphasising the pressing need for accurate, non-invasive and cost-effective early detection methods [1]. Molecular markers are among the most desirable diagnostic tools and have the potential to improve current detection power [2–4]. As opposed to cancer type-specific molecules, a single marker that allows for accurate detection of multiple common malignancies would be a powerful tool for early diagnosis or screening [5]. Theoretically, non-invasive detection of such a pan-cancer marker in bodily liquids or specimens acquired by minimally invasive procedures (bronchoalveolar lavage, nipple aspiration, ductal lavage etc.), could dramatically facilitate cancer detection and diagnosis. However, despite continuing interest in developing such pan-cancer diagnostic and screening tests, no satisfactory single marker for detection of different cancers has been introduced to the clinical applications as yet.

CpG methylation is a highly promising source of biomarker candidates [6], given the extensive reprogramming of every component of the epigenetic machinery including DNA methylation as cancer initiates and progresses [7, 8]. Alterations of DNA methylation patterns are (1) early events in virtually all tumor types [9], (2) more frequent than oncogenic mutations and affect genes/pathways characteristic of carcinogenesis in general [5], and (3) chemically stable and readily detectable in blood and other bodily liquids by current analytical methods [10]. Taken together, these observations strongly suggest that aberrations of DNA methylation may be instrumental for development of universal markers for detection of cancer at early stages.

In our earlier microarray-based search for a DNA methylation signature that is characteristic of breast cancer, hypermethylation of *growth hormone secretagouge receptor* (*GHSR*) exhibited high sensitivity and specificity of 89.3 and 100% for cancer detection, respectively [11]. Given this high level of accuracy for detection of breast carcinoma, we set out to verify whether *GHSR* methylation pattern is determinant of other malignancies. In this study, we report a *GHSR* methylation signature that is characteristic of multiple solid tumors and leukaemia. The degree of aberrant methylation enables accurate discrimination of cancer and respective control samples regardless of the tumor stage, and holds promise as a potential single marker for detection of multiple cancer types as a pan-cancer marker.

## RESULTS

### *GHSR* hypermethylation is a common epigenetic mark that distinguishes cancers from non-cancer specimens regardless of tumor type

**Figure 1:**
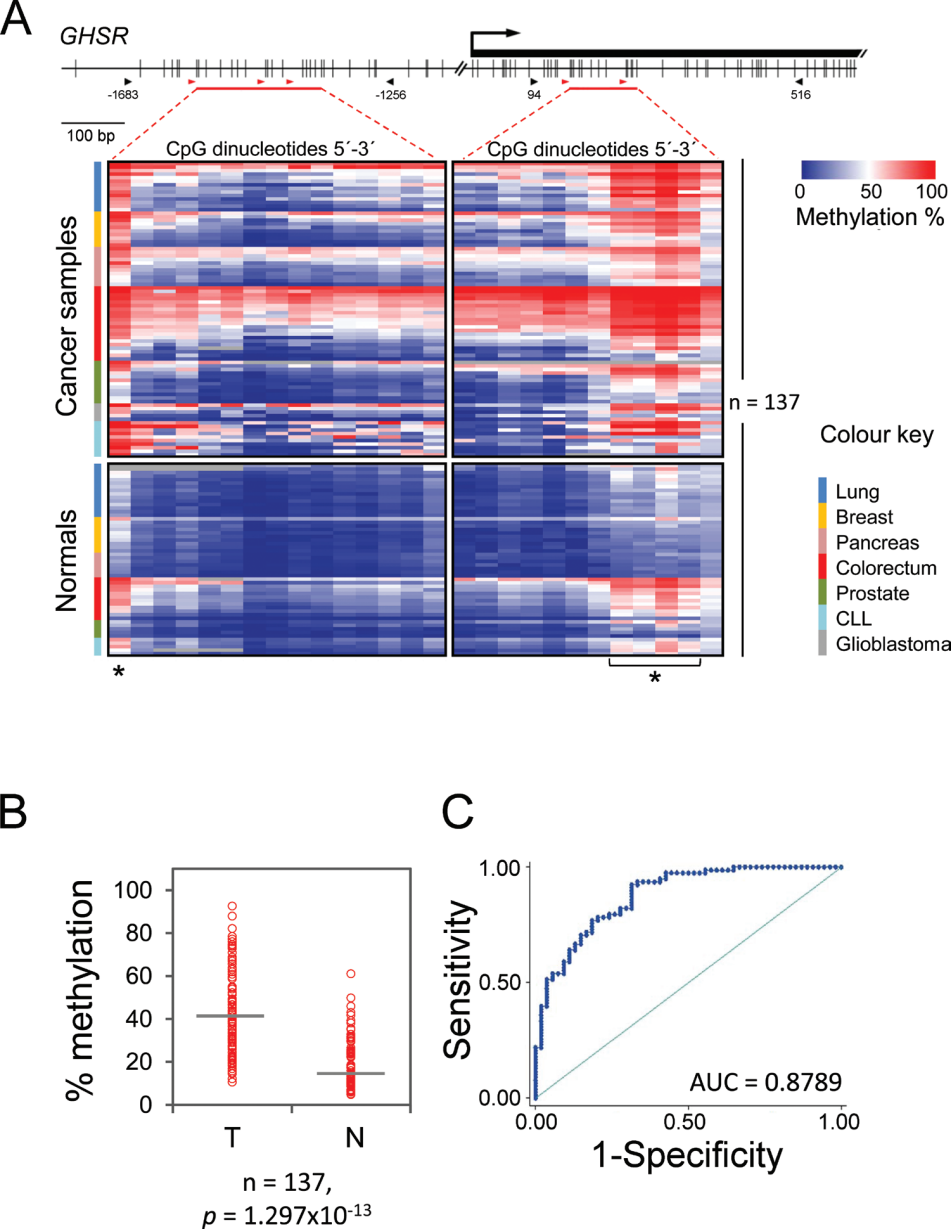
*GHSR* methylation pattern, average methylation degree and ROC curve analysis in seven common malignancies and respective normal tissue **(A)** CpG map of the interrogated regions (top). Vertical bars indicate the positions of CpG dinucleotides. The location of the first exon is shown as a black rectangle. The arrow indicates the *GHSR* transcriptional start site. The black arrowheads indicate the portions of the CpG islands within the gene's promoter and first exon that were PCR amplified (numbering relative to transcriptional start site); the horizontal red bars specify the CpG sites quantified by pyrosequencing; the red arrowheads denote the positions of sequencing primers. *GHSR* methylation pattern (bottom). Columns of the heatmap represent 27 CpG sites that are highlighted with horizontal red bars in the CpG map above. The degree of methylation was measured by bisulfite pyrosequencing in a total of 137 tissue specimens (shown as rows), which included cancer samples (top) of lung, breast, pancreas, colorectum, prostate, chronic lymphocytic leukaemia and glioblastoma as well as respective control tissue samples (bottom). Samples within each tumor entity are colour coded (legend at the right) and sorted descending by the average methylation degree. Asterisks denote CpG sites that were considered in ROC curve analysis. **(B)** Average percentage of methylation across 27 CpG sites (vertical axis) is plotted against the sample type (horizontal axis): tumor (T) and non-neoplastic tissue (N). Each circle indicates the methylation degree of a particular specimen. Horizontal bars denote the median methylation level for the cancer samples or the normal controls, respectively. For CLL, B cells of healthy donors were employed as control. *P*-value (Wilcoxon test) is shown. **(C)** ROC curve using average *GHSR* methylation across 27 CpG sites. The value of area under the curve (AUC) is shown.

To ascertain if *GHSR* hypermethylation is a common feature of a broad spectrum of cancers, bisulfite pyrosequencing was performed on a total of 137 specimens that represented seven common malignancies and respective normal tissue from cancer patients. Cancers of lung, prostate, pancreas, colorectum, breast, glioblastoma and B cell chronic lymphocytic leukaemia (CLL) were included in the study. A total of 27 CpG sites were accurately quantified including 15 CpG dinucleotides upstream of the transcriptional start site along with 12 CpG sites within the gene's first exon; the latter had been addressed in our earlier study [11] (Supporting Information Table 2 and Figure 1). Artificial variations introduced by PCR-bias and pyrosequencing process were corrected as described earlier [12]. Although methylation load was distributed unequally among CpG sites at both loci (Figure 1A), a substantially higher average methylation degree across all 27 CpG sites was recorded in all tumor samples as compared to the normal tissue samples collected from cancer patients (for solid cancers) or B cells of healthy individuals that served as control for CLL samples (Figure 1B). This observation suggests that aberrant *GHSR* hypermethylation is a common epigenetic alteration in different malignancies.

**Figure 2:**
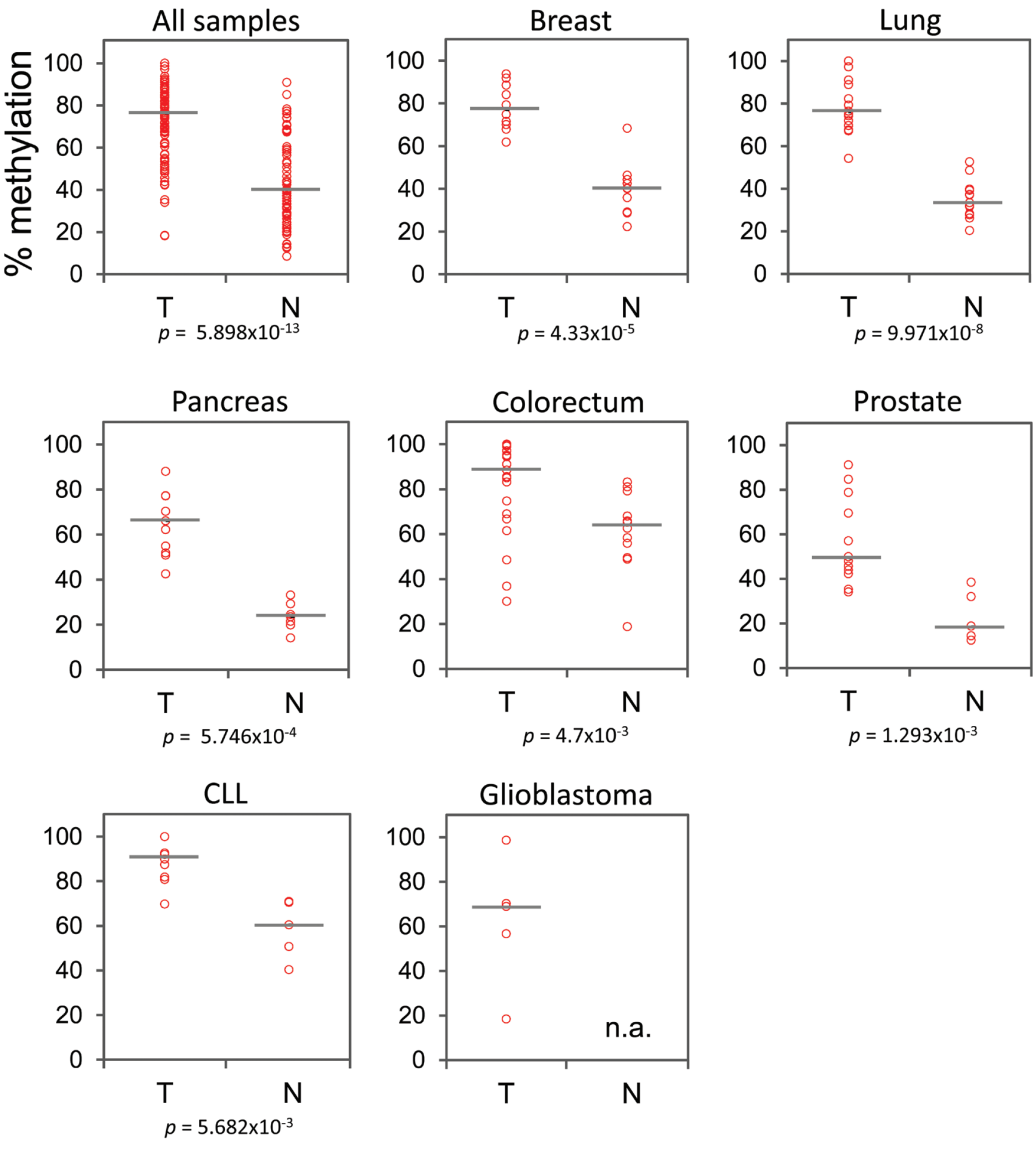
*GHSR* methylation degree in all samples analyzed Methylation of CpG *GHSR*_–1620 was analysed by bisulfite pyrosequencing. Percentage of methylation (vertical axis) is plotted against the sample type (horizontal axis): tumor (T) and normal-appearing tissue (N). Each circle indicates the methylation degree of a particular specimen. Horizontal bars denote the median methylation level for the cancer samples or the normal controls, respectively. For CLL, B cells of healthy donors were employed as control. Data are shown for the pooled specimens of seven malignancies as well as separately for each tumor type. The number of samples and *P*-values (Wilcoxon test) are additionally shown. Average of methylation levels of the 4 exonic CpGs (marked in Figure 1) is used for colorectal cancers.

### *GHSR* methylation signature: high specificity and sensitivity of cancer detection

We next assessed the accuracy of the methylation signature for detection of cancers by means of ROC analysis. The widely accepted criterion of accuracy is the area under the ROC curve, denoted as AUC. It may range in value from 0.5 (chance) to 1.0 (perfect discrimination or accuracy). We first performed ROC analysis on a total of 137 samples using the average methylation degree across all the 27 CpG sites that were interrogated in each sample. The analysis revealed a high degree of both sensitivity and specificity for discriminating cancers and respective non-neoplastic tissue specimens (AUC value of 0.8789, Figure 1C). An individual ROC analysis of each tumor type other than brain, for which no such analysis was possible due to the lack normal samples, resulted in high AUC values, too, particularly for cancers of pancreas, lung and breast as well as CLL with 1.0000, 0.9952, 0.9800 and 0.9400, respectively (Table 2). Detection accuracy was somewhat lower for cancers of prostate and colorectum with respective AUC values of 0.8833 and 0.8095.

Given the unequal distribution of DNA methylation load across the CpG sites, we next asked whether the discriminative power could be improved if specific CpG dinucleotides were taken into account. To this end, we identified a methylation signature of the CpG sites that showed markedly higher levels in cancer samples (*P* = 0.007, Grubbs' test). These were five CpG sites; one single site at –1620 position, and four consecutive CpGs at positions +249, +251, +257, +259 relative to *GHSR* transcription start site (Figure 1). Following testing methylation levels of each of these CpGs, methylation degree of the CpG located at –1620 position exhibited the most promising results for cancers of breast, prostate and CLL by increasing the detection accuracy slightly; 1.0000 vs. 0.9800, 0.9667 vs. 0.8833, 0.9600 vs. 0.9400 for these cancers, respectively (Supporting Information Figure 1). Although methylation patterns of –1620 CpG did not provide an improved AUC for detection of colorectal cancers, an average of methylation levels across CpG sites +249, +251, +257, +259 led to a stronger detection power for this cancer; AUC of 0.8492 vs 0.8095. Moreover, average methylation of the four exonic CpGs provided a stronger detection power than that of –1620 CpG for cancers of lung (0.9952 vs 0.9692). Detection power for the other cancers remained essentially the same (Table 2 and Supporting Information Figure 1).

### *GHSR* methylation degree is independent of the tumor progression and is detected at early tumor stages

**Figure 3:**
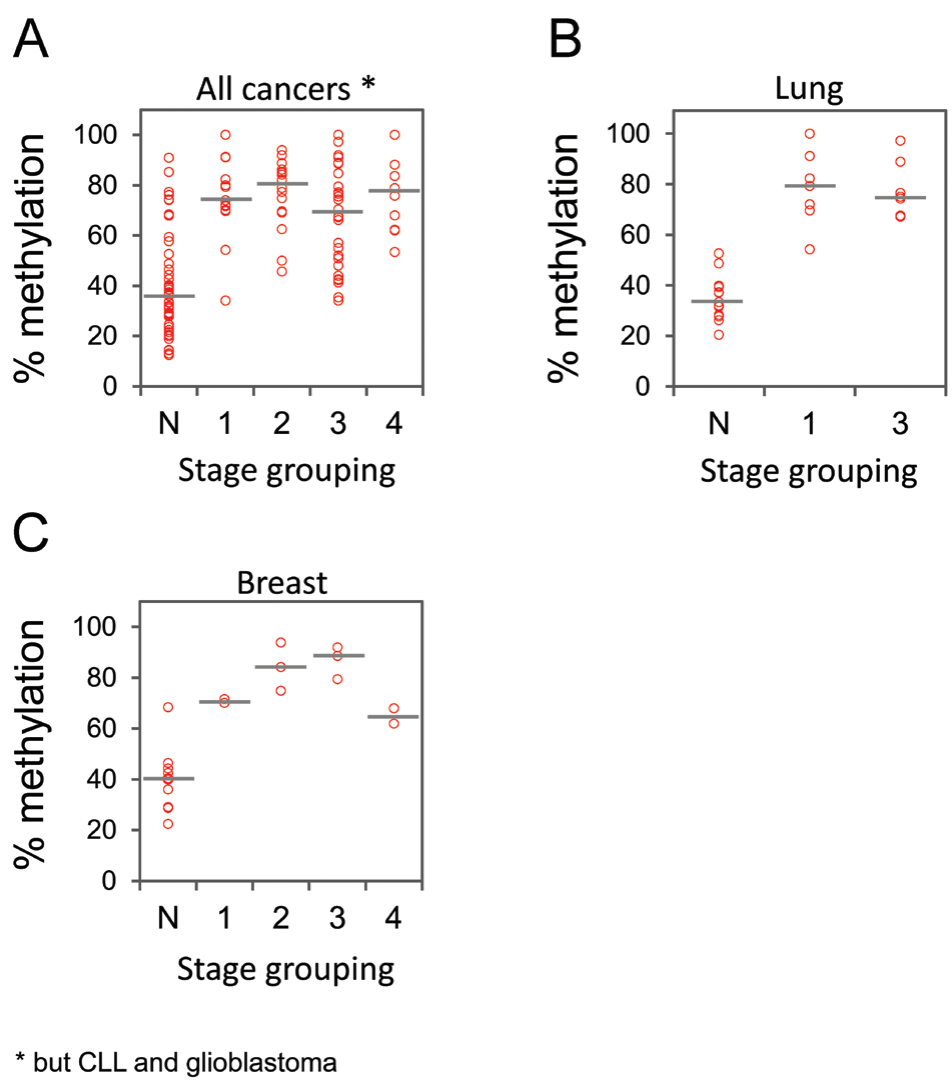
*GHSR* methylation in cancer samples of different stages Representative data are shown for the pooled specimens of cancers of breast, pancreas, lung, prostate and colorectum **(A)** as well as separately for lung **(B)** and breast **(C)** cancers. Percentage of methylation (vertical axis) is plotted against tumor stages (horizontal axis). Each circle indicates the methylation degree (CpG *GHSR*_–1620) of a particular tumor specimen. Horizontal bars denote the median methylation level for tumors of each stage.

Cancer specific aberrations of DNA methylation are considered as early events in process of tumor development [13]. We examined if the *GHSR* methylation signature can be detected at early stages of carcinogenesis and, therefore, holds promise for early cancer detection. While hypermethylated in tumors relative to control samples, no significant differences of *GHSR* DNA methylation degrees were observed across stages in pooled set of samples (Figure 3A), suggesting that *GHSR* methylation may be an early event in development of these cancers. This observation is in agreement with our previously reported findings that *GHSR* is hypermethylated in both invasive and non-invasive *in situ* ductal breast carcinomas [11]. Similarly, no differences in *GHSR* methylation were observed between different stages within each tumor entity for lung and breast cancers (Figure 3B–3C). We could not perform this analysis for other cancers due to the low number of tumors available in different stages.

### Validation of *GHSR* methylation signature in an independent set of pancreatic ductal adenocarcinomas

**Figure 4:**
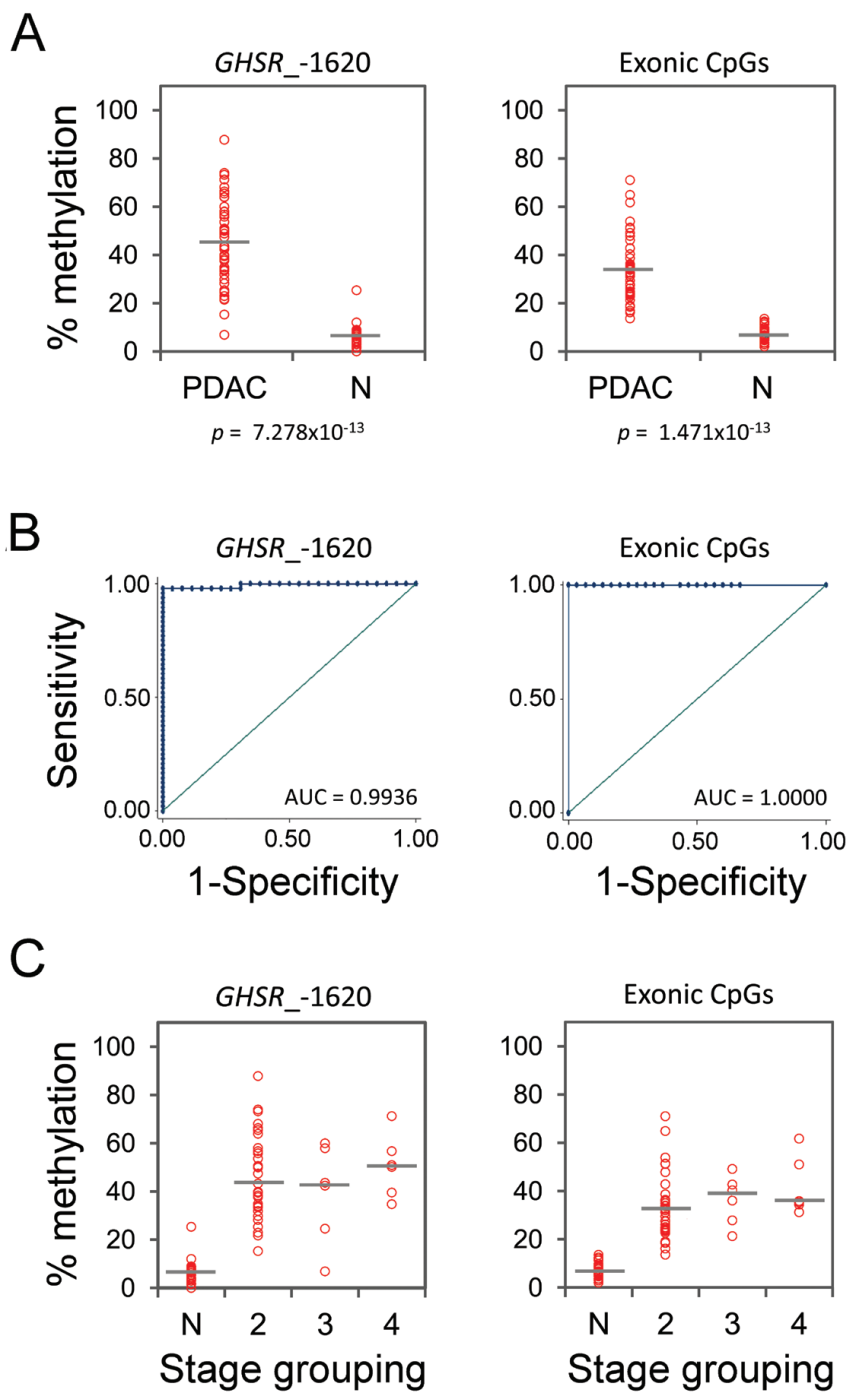
*GHSR* methylation degree, ROC curve analysis and methylation percentages across different stages in a validation set of PDAC and normal pancreas tissue specimens from healthy individuals **(A)** Percentage of methylation at CpG site *GHSR*_–1620 (left diagram) and average of methylation levels of the four exonic CpGs (right diagram, vertical axis) is plotted against the sample type (horizontal axis): PDAC and normal pancreas tissue (N). Each circle indicates the methylation degree of a particular specimen. Horizontal bars denote the median methylation level for the cancer samples or the normal controls, respectively. *P*-value (Wilcoxon test) is shown. **(B)** ROC curves using methylation percentages from **(A)**. The AUC values are shown. **(C)** Percentages of methylation from **(B)** (vertical axis) is plotted against tumor stages (horizontal axis). Each circle indicates the methylation degree of a particular tumor specimen. Horizontal bars denote the median methylation level for tumors of each stage. Note the essentially similar *GHSR* methylation levels at different stages of PDAC progression.

Having established the methylation signature for seven common malignancies by employing a limited number of specimens per tumor type, we set out to evaluate its discriminative power on a larger set of specimens. To this end, a validation set of 78 samples was used that comprised 48 PDAC along with 30 normal pancreas tissue specimens from healthy individuals. A total of 10 CpG sites were analysed by bisulfite pyrosequencing – six on the promoter including CpG site –1620 and the four exonic CpG dinucleotides. Corroborating our findings from the test samples, significant hypermethylation was detected at the signature CpGs (Figure 4A) in tumor specimens that accurately discriminated between PDAC and controls with AUC values of 0.9936 (95% CI: 0.96278–1.00000) and 1.0000 (95% CI: 1.0000–1.0000) corresponding to the CpG *GHSR*_–1620 and average methylation of the four exonic CpGs, respectively (Figure 4B). Moreover, the hypermethylation pattern was detected in both low and high stage tumors with no significant difference of methylation level between different stages (Figure 4C).

## DISCUSSION

In the current study, we aimed at identification of a DNA methylation signature that possesses a potential value as a marker of common cancers. This work is based on our earlier large scale DNA methylation study of breast tumors which led to the identification of *GHSR* hypermethylation as a highly accurate marker to discriminate cancers from normal breast tissue and benign lesions [11]. To test if *GHSR* methylation signature is of diagnostic value for other tumors, seven common malignancies were chosen, namely cancers of breast, lung, pancreas, prostate, colorectum as well as the most common lymphoproliferative disease CLL. In addition to the 12 CpG sites within the first exon's CpG island that had been interrogated earlier, we extended our analysis by examining additional 15 CpG dinucleotides within a promoter CpG island. Overall, significantly higher average degree of *GHSR* methylation across all 27 CpG sites was observed in all tumors than in normal controls. Cancers of breast, lung, pancreas, prostate and CLL were most accurately discriminated from respective normal controls. Importantly, high levels of aberrant methylation were detected already on early stages of all malignancies that were assessed. No significant differences were revealed across different stages suggesting that *GHSR* hypermethylation is an early cancer event. Of significance, these results were fully replicated in an independent validation set of 78 pancreatic adenocarcinomas and respective normal controls. Taken together, the facts that (1) *GHSR* is aberrantly hypermethylated in cancer specimens of different types, (2) its methylation levels accurately discriminate between cancer and control tissue specimens and (3) DNA methylation changes are detectable already in early stage tumors support a potentially high diagnostic value of *GHSR* methylation for a broad spectrum of malignancies.

For different cancers, higher or essentially the same detection accuracy was achieved if analysis was focused to only one single CpG site in the promoter region or four consecutive CpG units within the first exon. This narrowing down to a single or a few targeted CpG site(s) considerably simplifies the assay procedure, which can be performed in clinical setting by bisulfite pyrosequencing, methylation specific qPCR or genotyping assay such as MassArray of Sequenom. Obviously, as with any other biomarker candidate, a correct cut-off value for use in the clinic has to be defined in further multicentre validation studies. However, given the fact that tools for quantitative and high resolution analysis of DNA methylation – e.g., pyrosequencing or MassArray – has already become available in clinical laboratories, application of such threshold values as cancer biomarkers has become both technically and clinically feasible [8].

Interestingly, *GHSR* undergoes frequent aberrant hypermethylation regardless of both anatomic location – lung, breast, pancreas, colorectum – or histological origin – epithelial, haematopoietic or neuroendocrinal – of the malignancies that were interrogated. This finding is of importance because it suggests the possibility of identifying common epigenetic alteration acting across tumor types and presumably being at the root of carcinogenesis [5]. Although intrinsic susceptibility of *GHSR* locus to abnormal DNA methylation cannot be excluded, our earlier published data favour functionality of this epigenetic aberration by showing lower invasion of breast cancer cell line MDA-MB-231 following ectopic expression of *GHSR in vitro* [11]. These data indicate that epigenetic abrogation of *GHSR* expression may contribute to the pathogenesis of breast cancer. Further studies are warranted to ascertain the functional relevance of this epigenetic alteration for other malignancies and – if any – the molecular pathways mediating effect of *GHSR* deficiency on cancer cell behaviour.

Our study has some limitations. The limited number of samples per tumor type requires further validation on wider cohorts to confirm the diagnostic performance of the methylation signature reported here. Importantly, however, the fact that high diagnostic value of *GHSR* methylation is fully reproducible, for example, for breast cancer in the current study (*n* = 20), and in our earlier report [11] (*n* = 117) and for pancreatic ductal adenocarcinoma (test set *n* = 18 and validation set *n* = 78) suggests the high reproducibility of the signature for other cancers, too. Besides, detection of methylated *GHSR* alleles could not be tested in serum or other biological liquids in the current study, due to the lack of appropriate samples.

In conclusion, we report the DNA methylation signature of a single gene that is common to seven frequent malignancies, accurately discriminates cancer specimens from respective normal tissue samples and holds promise as a marker for early detection of a broad spectrum of cancers. The employment of this marker for clinical application needs to be evaluated by the clinical community. Assaying *GHSR* methylation may serve as a complementary approach to the current diagnostic methods for cancers that are difficult to detect early.

## MATERIALS AND METHODS

### Patient samples

A test set of 137 samples that represented seven different malignancies were employed in this study including cancers of lung, breast, prostate, pancreas, colorectum, glioblastoma and B cell chronic lymphocytic leukaemia (CLL) as well as normal tissue samples from cancer patients (Table 1). Fresh-frozen specimens of lung, prostate, colorectal adenocarcinomas and glioblastoma were obtained from the Lung Biobank of the Thoraxklinik Heidelberg and the tissue bank of the National Center for Tumor Diseases (NCT, Heidelberg, Germany). Samples of pancreatic ductal adenocarcinoma (PDAC) were provided by the European Pancreas Center (Heidelberg, Germany). Specimens of invasive ductal and ductal-lobular carcinoma of breast were obtained from Odessa State Medical University (Odessa, Ukraine). Blood samples of B cell chronic lymphocytic leukaemia were acquired from the National Research Centre for Haematology (Moscow, Russia). A validation set of 78 samples including PDAC specimens and normal pancreas tissue samples from healthy individuals were provided by the aforementioned source (Table 1). All the samples were obtained after ethical approval of the relevant review boards at the corresponding institutions; written informed consent had been obtained from all donors.

**Table 1:** Characteristics of patients and samples

Location	Lung		Prostate		Breast		Colorectum		Pancreas		Blood		Brain		Overall (test set)		Pancreas (validation)	
Specimens	T	N	T	N	T	N	T	N	T	N	T	N	T	N	T	N	T	N
Number	14	15	12	5	10	10	21	12	11	7	10	5	5	-	83	54	48	30
Sex																		
Male	10	9	12	5	-	-	13	6	9	5	2	0	-	-	46	25	31	18
Female	4	6	-	-	10	10	8	6	2	1	8	5	-	-	32	28	17	8
na	-	-	-	-	-	-	-	-	-	1	-	-	5	-	5	1	-	4
Age, years																		
Mean	61	53	64	63	60	60	56	61	63	53	49	38	na	-	59	55	64	49
Range	48–76	22–69	51–71	57–71	44–66	44–66	25–72	34–85	40–82	37–66	41–57	21–54	na	-	25–82	21–85	44–81	16–98
Stage grouping *																		
0	-	-	-	-	-	-	-	-	-	-	1	-	-	-	1	-	-	-
I	7	-	-	-	2	-	4	-	-	-	3	-	-	-	16	-	-	-
II	-	-	3	-	3	-	10	-	1	-	5	-	-	-	22	-	33	-
III	7	-	8	-	3	-	2	-	7	-	-	-	-	-	27	-	7	-
IV	-	-	1	-	2	-	3	-	3	-	1	-	-	-	10	-	7	-
na	-	-	-	-	-	-	2	-	-	-	-	-	5	-	7	-	1	-

T: Tumor; N: Normal; na: data not available

* For CLL, Rai staging is shown [15].

**Table 2:** Crude and adjusted area under the ROC curve (AUC) values by cancer site

Area under the ROC curve (AUC). Cancer vs. normal tissue*										
	Average methylation percentage across all 27 CpGs				Methylation percentage of GHSR_−1620			Average methylation percentage across four most hypermethylated consecutive CpGs in exon 1**		
Malignancy	*n*	Crude	(95% CI)	Age-adjusted	Crude	(95% CI)	Age-adjusted	Crude	(95% CI)	Age-adjusted
Breast	20	0.9400	0.8181–1.0000	0.9800	0.9800	0.9329–1.0000	1.0000	0.9900	0.9623–1.0000	1.0000
Lung	29	0.9905	0.9679–1.0000	0.9952	1.0000	1.0000–1.0000	0.9692	0.9762	0.9263–1.0000	0.9952
Pancreas	18	1.0000	1.0000–1.0000	1.0000	1.0000	1.0000–1.0000	1.0000	1.0000	1.0000–1.0000	1.0000
Colorectum	33	0.7976	0.6406–0.9546	0.8095	0.6726	0.4815–0.8637	0.7341	0.8016	0.6469–0.9562	0.8492
Prostate	17	0.8667	0.6320–1.0000	0.8833	0.9667	0.8879–1.0000	0.9667	0.8909	0.6683–1.0000	0.8909
CLL	15	0.9400	0.8123–1.0000	NC	0.9600	0.8681–1.0000	NC	0.5600	0.2420–0.8779	NC
All cancers	132	0.8789	0.8206–0.9372	0.8862	0.8640	0.8001–0.9280	0.8776	0.8759	0.8177–0.9341	0.8907

NC: Not calculable. Brain samples were not included in the analysis due to the lack of normal tissues.

* For CLL, B cells of healthy donors were employed as control.

** *GHSR*_+249, *GHSR*_+251, *GHSR*_+257, *GHSR*_+259 were considered

### DNA isolation and bisulfite conversion

DNA was extracted from samples using DNeasy Blood & Tissue kit (Qiagen, Hilden, Germany) as instructed by the manufacturer. The quantity of DNA was measured with a ND-1000 spectrophotometer (Thermo Scientific, Wilmington, USA). A total of 2.0 μg DNA was treated with sodium bisulfite using the EpiTect Bisulfite kit (Qiagen).

### PCR amplification

PCR amplification of the target regions at the first exon of GHSR (described in [11] and indicated in Fig.1) was carried out in 25 μl reactions of 1 μl bisulfite-converted DNA, 1.5 mM MgCl2, 125 mM dNTP, 200 nM primers, 0.65 units HotStar Taq DNA polymerase and 1x Q-solution (Qiagen). An amplification programme was used as described earlier [11]. Briefly, amplification started by an initial activation of the HotStar Taq DNA polymerase at 95°C for 15 min. The first amplification cycle was denaturation at 95°C for 1 min, annealing at 60°C for 2 min and elongation at 72°C for 2 min. This procedure was continued for 7 cycles, reducing the annealing temperature by 1°C at each cycle, followed by 38 cycles of 1 min denaturation at 95°C, 2 min annealing at 52°C and 2 min elongation at 72°C. The target promoter region was amplified without addition of Q-solution by employing a similar programme with 64°C as starting annealing temperature that was reduced by 1°C at each cycle for 7 cycles, followed by 38 cycles with annealing temperature of 56°C. The sequences of the PCR primers are listed in Supporting Information Table 1. About 5 μl of each reaction was examined on 2% agarose gels.

### Bisulfite pyrosequencing

A volume of 20 μl of each PCR product was mixed with 2 μl Streptavidin Sepharose High Performance (GE Healthcare, Uppsala, Sweden), 38 μl of PyroMark binding buffer and 10 μl water. The PyroMark Q24 Vacuum Workstation (Qiagen) was used to prepare single-stranded DNA. The Sepharose beads with the single-stranded templates attached were released into a PyroMark Q24 Plate (Qiagen) containing 25 μl of 0.3 μM corresponding sequencing primer in annealing buffer. Pyrosequencing reactions were carried out using the PyroMark Gold Q24 Reagents (Qiagen) in a PyroMark Q24 Pyrosequencing System (Qiagen) according to the manufacturer's protocol. The sequences of the pyrosequencing primers are listed in Supporting Information Table 1. Quantification of CpG methylation was performed using the software PyroMark Q24 v.2.0.6 (Qiagen). The moderate amplification bias towards unmethylated alleles was corrected using the calibration data derived from a set of control samples and cubic polynomial regression as previously described [12].

### Receiver operating characteristic (ROC) curves

Using Stata statistical package (version 8), the area under the ROC curve [AUC; ranging from 0.5 (chance) to 1.0 (perfect discrimination or accuracy)] was measured to characterise the accuracy of DNA methylation signature to discriminate the malignant tissues from control samples. Stata ‘lroc’ command was used after logistic regression analyses to produce adjusted ROC curves.

### Statistical tests

The average methylation percentage was calculated across the CpG sites that were interrogated. Differences in the degree of methylation between cancer specimens and respective control samples were determined by Wilcoxon rank sum test using an R computing environment [14]. *P*-values of less than 0.05 were regarded as statistically significant.

## SUPPLEMENTARY FIGURE AND TABLES


