# Supplementary material for: GHSR DNA hypermethylation is a common epigenetic alteration of high diagnostic value in a broad spectrum of cancers
Source: Oncotarget. 2015 Feb 21;6(6):4418–27. doi: 10.18632/oncotarget.2759 (PMC4414200; doi:10.18632/oncotarget.2759)
Supplement: Supplementary file 1 [file oncotarget-06-4418-s001.pdf]

SUPPLEMENTARY FIGURE AND TABLES

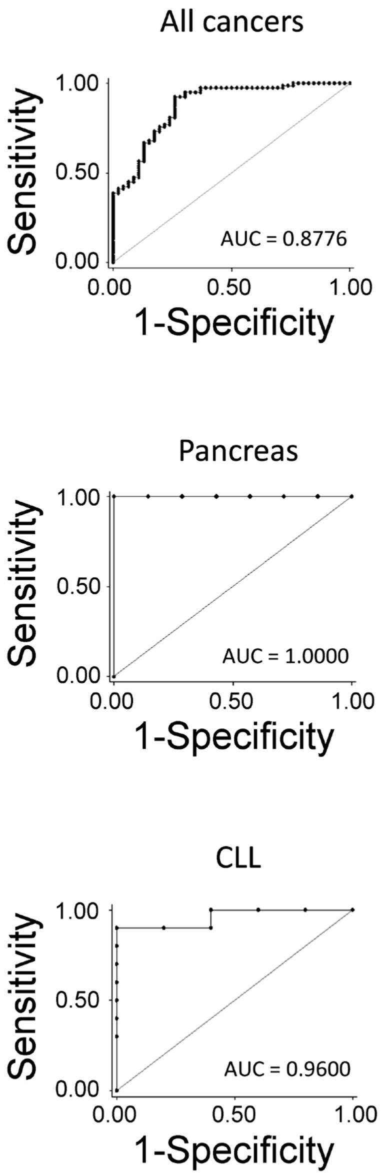

Supporting Information Figure 1: ROC curves using *GHSR* methylation. Cancers were compared with normal-appearing tissue samples for pooled 137 specimens as well as separately for each tumor entity. Methylation degree of CpG *GHSR*_−1620 was used for calculations for all cancers but colorectal and lung cancers for which average of methylation levels of the 4 exonic CpGs (marked in Figure 1) is used.

Supporting Information Table 1: Sequences of the PCR and pyrosequencing primers used in this study

Gene symbol	Primer sequences (5′-3′)<br>F: PCR forward; R: PCR reverse;<br>S: pyrosequencing; bio: biotinylation;<br>up: upstream; dwn: downstream CGI	Amplicon length, bp	Number of CpGs quantified by pyrosequencing
GHSR-up-F	ATTGTTTGTGAAAGGTAAGTATAAA	427	
GHSR-up-R	bio-ACACTAACAAAATAACCCTCTAAAC		
GHSR-up-S1	GTATTTAATTGAGGGGGA		6
GHSR-up-S3	GTGGGAGTTTGTTTTGTT		4
GHSR-up-S6	GTGATAGGGAAGTTATTTTTTTAG		5
GHSR-dwn-F	GATTTGGATTGGGATGTTTTTTT	422	
GHSR-dwn-R	bio-CCCTTAATAACCACCACCTTAAC		
GHSR-dwn-S2	ATTATGTTGGTGGTGT		5
GHSR-dwn-S4	GAGTTGTTGTAGT		7

Supporting Information Table 2: CpG sites of *GHSR* interrogated by bisulfite pyrosequencing

CpG ID	Sequencing primer	Distance to TSS*
GHSR_–1620	S1	–1620
GHSR_–1607		–1607
GHSR_–1599		–1599
GHSR_–1596		–1596
GHSR_–1568		–1568
GHSR_–1565		–1565
GHSR_–1530	S3	–1530
GHSR_–1512		–1512
GHSR_–1503		–1503
GHSR_–1490		–1490
GHSR_–1458	S6	–1458
GHSR_–1455		–1455
GHSR_–1448		–1448
GHSR_–1431		–1431
GHSR_–1399		–1399
GHSR_+160	S4	+160
GHSR_+162		+162
GHSR_+165		+165
GHSR_+174		+174
GHSR_+178		+178
GHSR_+193		+193
GHSR_+205		+205
GHSR_+249	S2	+249
GHSR_+251		+251
GHSR_+257		+257
GHSR_+259		+259
GHSR_+266		+266

* TSS: transcriptional start site according to the Database of Transcriptional Start

SUPPORTING INFORMATION REFERENCE

1. Yamashita R,Sathira NP, Kanai A, Tanimoto K, Arauchi T,Tanaka Y, Hashimoto S, Sugano S, Nakai K, Suzuki. Genome-wide characterization of transcriptional start sites in humans by integrative transcriptome analysis. Genome Res. 2011; 21:775–789.
